# Supplementary material for: Metabolic and chromosomal changes in a Bacillus subtilis whiA mutant
Source: Microbiol Spectr. 2023 Nov 2;11(6):e01795-23. doi: 10.1128/spectrum.01795-23 (PMC10714963; doi:10.1128/spectrum.01795-23)
Supplement: Supplemental material — Tables S1 to S5 and Fig. S1 to S5. [file spectrum.01795-23-s0001.pdf]

Supporting information

Metabolic and chromosomal changes in a *Bacillus subtilis whiA* mutant

Laura C. Bohorquez, Joana de Sousa, Transito Garcia-Garcia, Gaurav Dugar, Biwen Wang, Martijs J. Jonker, Marie-Françoise Noirot-Gros, Michael Lalk, Leendert W. Hamoen

Content:

- Table S1: Composition of different chemically defined media for *B. subtilis*
- Table S2: Transcription response of the main genes involved in branched-chain amino acid and fatty acid metabolism.
- Table S3: Fatty acid profile of the Δ*whiA* mutant
- Table S4: Membrane fluidity analysis using Laurdan generalized polarization
- Table S5: *B. subtilis* strains used in this study
- Fig. S1: Principal Component Analysis (PCA) of the exometabolome
- Fig. S2: Loading plots for PCA analysis depicted in Fig. S1
- Fig. S3: Growth of a Δ*whiA* strain in Amber medium with different carbon sources
- Fig. S4: Correlation with a previous transcriptome studies of Δ*whiA* grown in LB medium
- Fig. S5: Main fatty acid synthesis pathways and related genes in *B. subtilis*
- References

Table S1. Composition of different chemically defined media for *B. subtilis*

medium	M9	CSE-G	MOPS-MM	minimal sporulation	minimal competence	SMM based	Belitzky MM/BLM	Chemostat	High phosphate	AMBER
Refs	(1)	(2)	(3)	(4)	(5)	(6)	(7)	(8)	(8)	This study
Doubling time	55-70 min	43 min	50 min	NA	NA	120 min	NA	NA	NA	50 min
Issues	lysis in stat phase	NA	linear growth from OD 0.9	High Mg to prevent lysis	NA	NA	NA	NA	NA	NA
**Components (mM)**										
K_2HPO_4		70	~ 2	0.5	78	80				70
Na_2HPO_4	48							5		
KH_2PO_4	22	30	~ 0.9		42.8	44	0.6		3.5	30
Tris							50		50	
K-MOPS			40							
$(NH_4)_2SO_4$		25	15		14.7	15	15	50	3	10
NH_4Cl	18.7			10						
NH_4NO_3				1.2						
KCl							27			
NaCl	17.11									15
Na_2SO_4				0.8						
K_2SO_4								30		
$MgSO_4$	1	0.5	0.811	20	6.6	2.7	8		3.5	1
$MgCl_2$				0.009				0.004		
$CaCl_2$	0.1			1		0.05	2	0.1		0.1
$MnSO_4$		0.01		0.01		0.001	0.01			0.002
$MnCl_2$	0.005		0.00008					0.025	1	
Fe-NH_4-citrate		0.084			0.0042					0.01
$FeCl_3$	0.05		0.005	0.001				0.1	3	
$FeSO_4$							0.001			
$ZnCl_2$	0.0125	0.0125	0.00001					0.025	0.01	0.002
$CuSO_4$			0.00001							
$CuCl_2$	0.0025							0.005		0.002
$CoCl_2$	0.0025		0.00003					0.005		0.002
Na_2MoO_4	0.0025							0.005		0.002
tryptophan		0.25	0.25		0.095		0.78	1		
Na_2 succinate		37								
K glutamate		43		10		10	4.5	5		10
glucose	16.7	5.5	27.5	11	26.9	11	11	(glycerol)	28	22
Na_3 citrate					3.3	3.4	7	1	7	
PEP (13 amino acids)					10 mg/l				CAS 0.05% + arginine 10mM	
K	22	213	45	11	209	214	32	65	3.5	180
Na	113	74		1	10	10	21	10	21	15
Mg	1	0.5	0.8	20	7	3	8	0.004	3.5	1
Fe	0.05	0.084	0.005	0.001	0.004		0.001	0.1	3	0.01
Ca	0.1			1		0.05	2	0.1		0.1
Mn	0.005	0.01	0.00008	0.01		0.001	0.01	0.025	1	0.002
Zn	0.0125	0.0125	0.00001					0.025	0.01	0.002
Cu	0.0025		0.00001					0.005		0.002
Co	0.0025		0.00003					0.005		0.002
Mo	0.0025							0.005		0.002
NH_4	19	50	30	11	29	30	30	100	6	20

PO_4	70	100	3	0.5	121	124	1	5	3.5	100
Cl	36	0.025	0.015	12		0.05	31	0.5	11	15
SO_4	1	26	0.8	21	21	18	23	80	6.5	11
succinate		37								
glutamate		43		10		10	5	5		10
citrate					3	3	7	1	7	
glucose	17	6	28	11	27	11	11		28	22
total (mM)	279	550	108	99	427	423	171	267	94	374

Table S2. Transcription response of the main genes involved in branched-chain amino acid and fatty acid metabolism
The main fatty acid synthesis pathways are shown in Fig. S5. Wild-type (strain 168) and *whiA* marker-less mutant (strain KS696) were grown in defined minimal Amber medium with glucose and malate and harvested for RNA isolation during exponential growth (OD_{500} ~0.5). Genes with an adjusted *p*-value < 0.05 are printed bold. Fold Change (FC) measured as Δ*whiA*/wt.

Gene	FC	*p*-value	Function
yvbW	-1,7	0,00039	leucine permease
braB	1,4	2,E-02	branched-chain amino acid
bcaP	1,9	2,E-02	branched-chain amino acid
brnQ	1,1	0,88972	branched-chain amino acid
azlC	1,1	0,8265	leucine permease
azlD	-1,0	0,96116	leucine permease
ilvA	-1,0	0,91403	biosynthesis of branched-chain
ilvB	-1,1	0,63163	biosynthesis of branched-chain
ilvH	1,2	0,67524	biosynthesis of branched-chain
ilvC	-1,3	0,09376	biosynthesis of branched-chain
leuA	-1,3	3,E-02	biosynthesis of leucine
leuB	-1,4	5,E-03	biosynthesis of leucine
leuC	-1,4	3,E-03	biosynthesis of leucine
leuD	-1,3	3,E-02	biosynthesis of leucine
ilvD	1,5	1,E-07	biosynthesis of branched-chain
ybgE (ilvE)	1,9	3,E-02	biosynthesis of branched-chain
ywaA (ilvK)	1,0	0,86738	biosynthesis of branched-chain
bkdR	1,4	0,14223	regulation of branched-chain
bcd	1,1	0,89767	utilization of branched-chain
lpdV	1,3	0,35449	utilization of branched-chain
bkdAA	1,4	0,09679	utilization of branched-chain
bkdAB	1,4	0,15037	utilization of branched-chain
bkdB	1,3	0,1743	utilization of branched-chain
ptb	-1,0	0,90811	utilization of branched-chain
buk	1,2	0,43644	utilization of branched-chain
bkdAA	1,4	0,09679	utilization of branched-chain
bkdAB	1,4	0,15037	utilization of branched-chain
bkdB	1,3	0,1743	utilization of branched-chain
buk	1,2	0,43644	utilization of branched-chain
ptb	-1,0	0,90811	utilization of branched-chain
mmgA	-1,5	0,99	utilization of branched-chain
mmgB	1,0	0,99	utilization of branched-chain
mmgC	1,3	0,99	utilization of branched-chain
mmgD	-1,8	0,33092	utilization of branched-chain
mmgE	-1,2	0,79287	utilization of branched-chain
mmgF	1,1	0,76859	utilization of branched-chain
yngE	-1,2	0,82102	utilization of branched-chain
yngF	1,1	0,99	utilization of branched-chain
yngG	-1,4	0,66001	utilization of branched-chain
yngHA	-1,2	0,81615	utilization of branched-chain
yngB	-1,1	0,83456	utilization of branched-chain

yngHB	-1,0	0,99	utilization of branched-chain
yngI	1,2	0,83368	utilization of branched-chain
yngJ	1,3	0,7173	utilization of branched-chain
fadN	-3,9	3,E-03	fatty acid degradation
fadA	-3,4	8,E-03	fatty acid degradation
fadE	-3,5	6,E-03	fatty acid degradation
lcfA	-1,1	0,73048	fatty acid degradation
fadR	-1,8	0,11584	regulation of fatty acid
fadB	-1,9	0,1207	fatty acid degradation
etfB	-2,5	2,E-02	fatty acid degradation
etfA	-1,7	0,15959	fatty acid degradation
lcfB	-1,5	0,38477	fatty acid degradation
acdA	-1,7	0,24359	fatty acid degradation
rpoE	-1,1	0,6558	fatty acid degradation
fatR (yrhI)	3,1	6,E-09	regulation of fatty acid
yrhJ	3,1	5,E-10	fatty acid degradation

Table S3. Fatty acid profile of the Δ*whiA* mutant

Fatty acids were analyzed as fatty acid methyl esters using gas chromatography. Fatty acid distribution is presented as mean and standard deviation of triplicate measurements.

Fatty acids	p-value	Mean of wild type	Mean of Δ*whiA*	difference	SE of difference
$C_{13:0}$ iso	0.0123	0.1400	0.0900	0.0500	0.0116
$C_{13:0}$ anteiso	0.5034	0.4900	0.5700	-0.0800	0.1089
$C_{14:0}$ iso	0.0037	3.9900	3.2130	0.7767	0.1277
$C_{14:0}$	0.0169	0.3100	0.3867	-0.0767	0.0194
$C_{15:0}$ iso	0.0001	21.8000	16.1600	5.6470	0.3697
$C_{15:0}$ anteiso	0.0010	29.7000	33.3800	-3.6770	0.4299
C15:0	0.0001	0.1433	0.3067	-0.1633	0.0111
$C_{16:0}$ iso	0.4350	13.1700	12.8500	0.3200	0.3692
$C_{16:0}$	0.0017	3.3470	4.8000	-1.4530	0.1938
$C_{17:0}$ iso	0.0189	14.3900	13.1500	1.2400	0.3251
$C_{17:0}$ anteiso	0.0004	9.9470	13.3200	-3.3700	0.3050
C17:0	0.0550	0.1067	0.2067	-0.1000	0.0373
$C_{18:0}$ iso	0.0317	0.3000	0.3700	-0.0700	0.0216
$C_{18:0}$	0.3191	0.7233	0.8767	-0.1533	0.1349
Others	0.3597	1.4400	0.3267	1.1130	1.0770

Table S4. Membrane fluidity analysis using Laurdan generalized polarization

Wild type and *whiA* mutant (KS400) strains were grown to an OD_{600} of approximately 0.5 in LB or Spizizen minimal salt medium (SMM). The membrane fluidity was measured by Laurdan generalized polarization (Laurdan GP) and the values were calculated as reported before (9), and presented as mean and standard deviation of 3 independent measurements. As a positive control the membrane fluidity was increased (decrease in Laurdan GP) by addition of 30 mM membrane fluidizer benzyl alcohol (BA) (9).

	wild type		Δ*whiA*	
Laurdan GP (LB)	0.608	±0.001	0.611	±0.001
Laurdan GP with BA (LB)	0.563	±0.009		
Laurdan GP (SMM)	0.632	±0.007	0.646	±0.002
Laurdan GP with BA (SMM)	0.592	±0.009	0.580	±0.012

Table S5. ***B. subtilis*** **strains used in this study**

Mutants kindly provided by other labs were transformed into our laboratory wild-type strain to ensure isogenic backgrounds.

Strain	Genotype	Reference
Wild type 168	*trpC2*	Lab stock
LB45	*zapA::tet whiA:Pspac-whiA(erm) aprE::lacI (spc)*	(10)
KS696	*markerless ΔwhiA mutant*	(11)
KS400	*whiA::km*	(11)

Fig. S1.

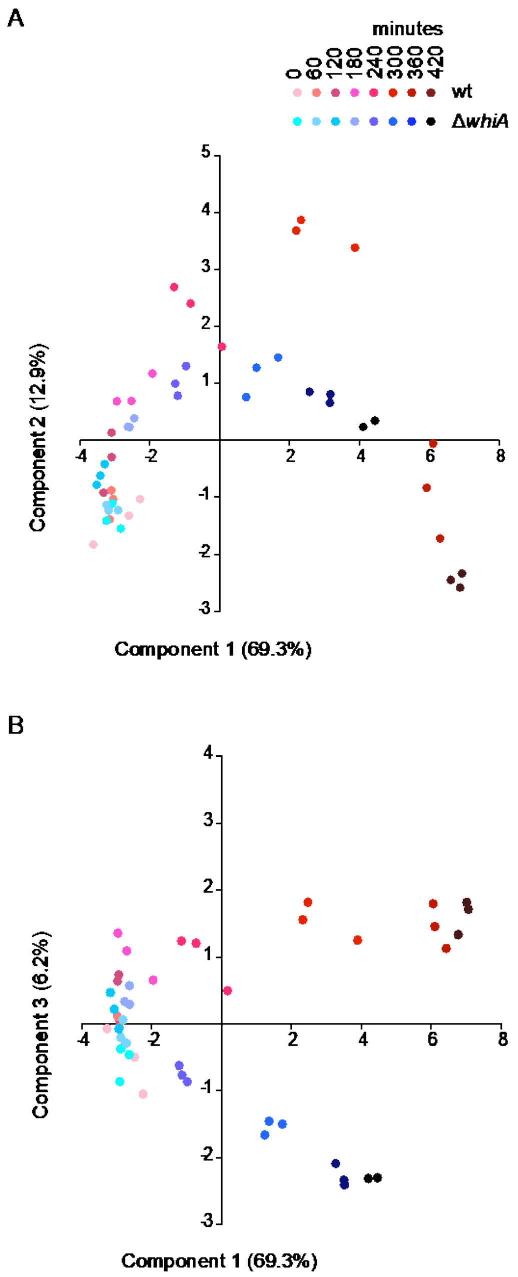

Fig. S1. Principal Component Analysis (PCA) of the exometabolome
The calculated extracellular metabolite concentrations of wild-type (strain 168) and *whiA* marker-less mutant (strain KS696), grown in defined minimal Amber medium with glucose and malate in this case, were log transformed, mean centered, autoscaled and applied to a principal component analysis. The plots display (A) principal component 1 versus principal component 2, and (B) principal component 1 versus principal component 3, with their corresponding proportion of variation. Loading values used are shown in the next figure (Fig. S2). Single values of 3 biological replicates are displayed. Groups indicated by the same color correspond to the same time point.

Fig. S2.

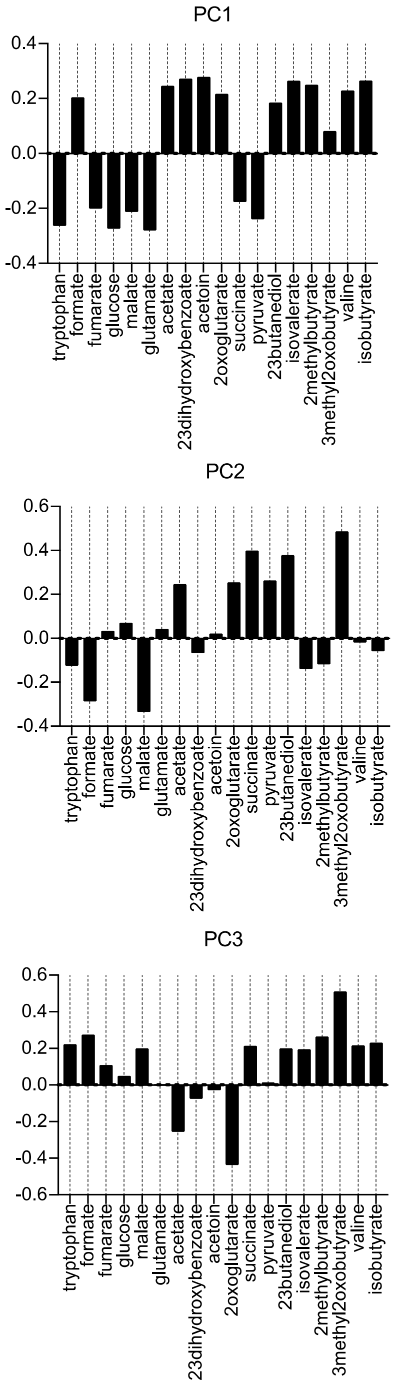

Fig. S2. Loading plots for PCA analysis depicted in Fig. S1

The calculated extracellular metabolite concentrations of wild-type (strain 168) and the *whiA* marker-less mutant (strain KS696) grown in defined minimal Amber medium with glucose and malate (22 mM each), were log transformed, mean centered, autoscaled, and applied to principal component analysis. Loading plots of component 1, 2 and 3 are displayed. The PCA plots are shown in Fig. S1.

Fig. S3.

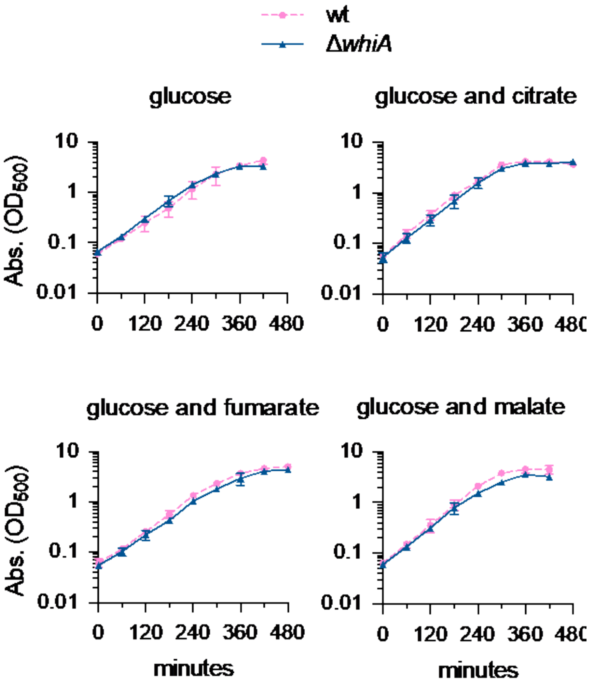

Fig. S3. Growth of a Δ*whiA* strain in Amber medium with different carbon sources
Growth of the wild-type strain (strain 168) and the *whiA* marker-less mutant (strain KS696) in chemically defined minimal Amber medium supplemented with 22 mM of either glucose, glucose and citrate, glucose and fumarate or glucose and malate. Data are shown as mean values and standard deviation of triplicate samples.

Fig. S4.

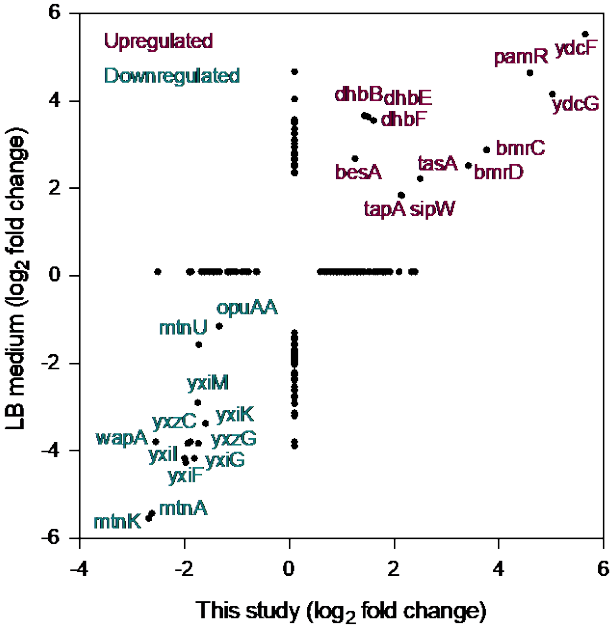

Fig. S4. Correlation with a previous transcriptome studies of Δ*whiA* grown in LB medium
Comparison of differently expressed genes in the *whiA* mutant grown in either LB medium (11) or defined minimal Amber medium supplemented with glucose and malate (this study). Only the most significantly up and down-regulated genes were compared. Correlated upregulated and downregulated genes are indicated in red and green, respectively.

Fig. S5.

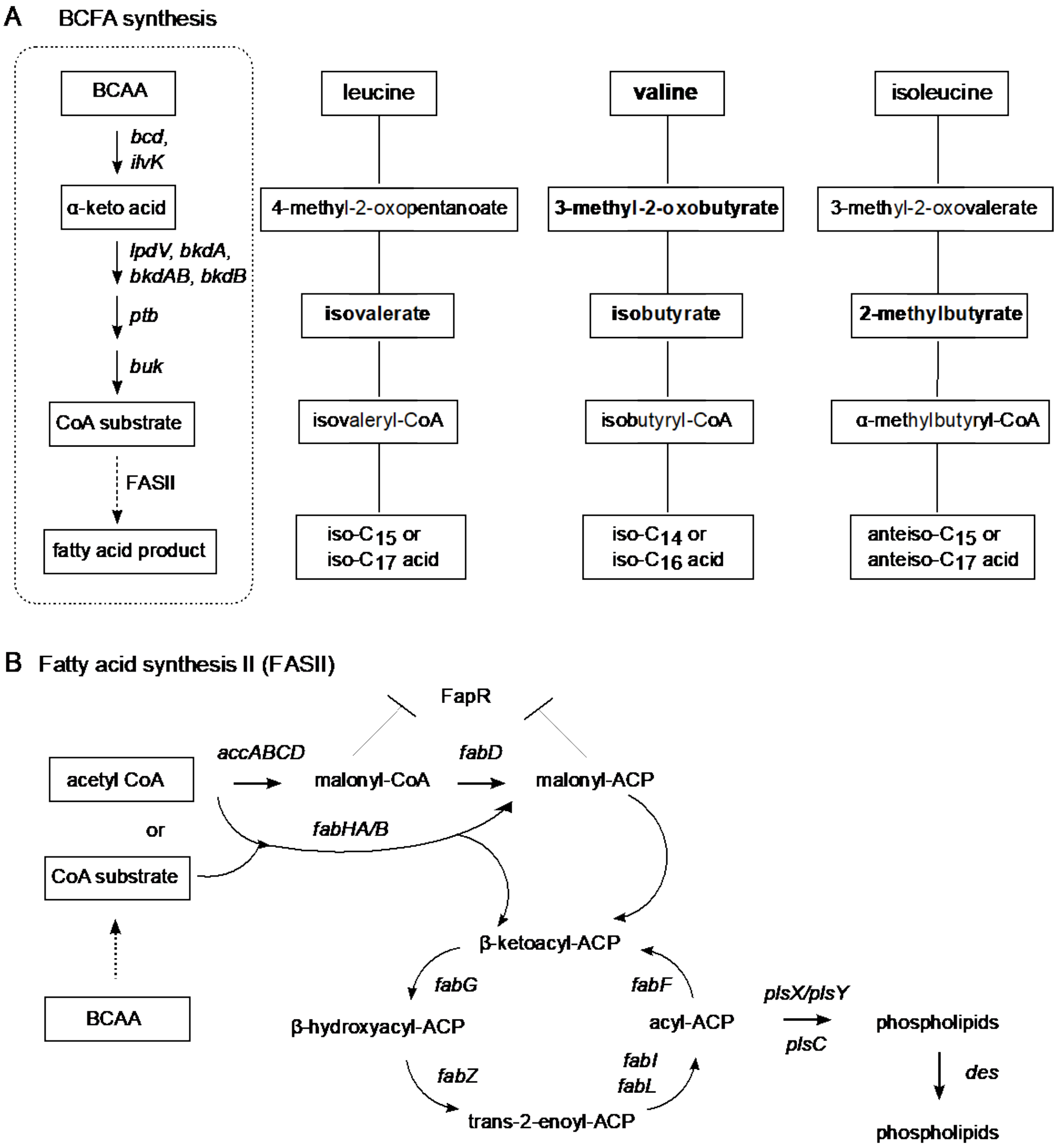

Fig. S5. Main fatty acid synthesis pathways and related genes in *B. subtilis*
(A) Branched-chain fatty acid precursor synthesis and main genes involved (12, 13). (B) Main fatty acid synthesis (FASII) pathway and main genes involved (14).

References

1. Kleijn RJ, Buescher JM, Le Chat L, Jules M, Aymerich S, Sauer U. 2010. Metabolic fluxes during strong carbon catabolite repression by malate in *Bacillus subtilis*. J Biol Chem 285:1587-96.
2. Wacker I, Ludwig H, Reif I, Blencke HM, Detsch C, Stulke J. 2003. The regulatory link between carbon and nitrogen metabolism in *Bacillus subtilis*: regulation of the *gltAB* operon by the catabolite control protein CcpA. Microbiology (Reading) 149:3001-3009.
3. Chen L, James LP, Helmann JD. 1993. Metalloregulation in *Bacillus subtilis*: isolation and characterization of two genes differentially repressed by metal ions. J Bacteriol 175:5428-37.
4. Thomaides HB, Davison EJ, Burston L, Johnson H, Brown DR, Hunt AC, Errington J, Czaplewski L. 2007. Essential bacterial functions encoded by gene pairs. J Bacteriol 189:591-602.
5. van Sinderen D, Venema G. 1994. *comK* acts as an autoregulatory control switch in the signal transduction route to competence in *Bacillus subtilis*. J Bacteriol 176:5762-70.
6. Spizizen J. 1958. Transformation of biochemically deficient strains of *Bacillus subtilis* by deoxyribonucleate. Proc Natl Acad Sci U S A 44:1072-8.
7. Stulke J, Hanschke R, Hecker M. 1993. Temporal activation of beta-glucanase synthesis in *Bacillus subtilis* is mediated by the GTP pool. J Gen Microbiol 139:2041-5.
8. Muler JP, An Z, Merad T, Hancock IC, Harwood CR. 1997. Influence of *Bacillus subtilis phoR* on cell wall anionic polymers. Microbiology (Reading) 143 (Pt 3):947-956.
9. Strahl H, Burmann F, Hamoen LW. 2014. The actin homologue MreB organizes the bacterial cell membrane. Nat Commun 5:3442.
10. Bohorquez LC, Surdova K, Jonker MJ, Hamoen LW. 2018. The Conserved DNA Binding Protein WhiA Influences Chromosome Segregation in *Bacillus subtilis*. J Bacteriol 200.
11. Surdova K, Gamba P, Claessen D, Siersma T, Jonker MJ, Errington J, Hamoen LW. 2013. The conserved DNA-binding protein WhiA is involved in cell division in *Bacillus subtilis*. J Bacteriol 195:5450-60.
12. Kaneda T. 1977. Fatty acids of the genus Bacillus: an example of branched-chain preference. Bacteriol Rev 41:391-418.
13. Michna RH, Zhu B, Mader U, Stulke J. 2016. SubtiWiki 2.0 - an integrated database for the model organism *Bacillus subtilis*. Nucleic Acids Res 44:D654-62.
14. Diomande SE, Nguyen-The C, Guinebretiere MH, Broussolle V, Brillard J. 2015. Role of fatty acids in Bacillus environmental adaptation. Front Microbiol 6:813.
